# Optically modulated magnetic resonance of erbium implanted silicon

**DOI:** 10.1038/s41598-019-55246-z

**Published:** 2019-12-13

**Authors:** Mark A. Hughes, Heqing Li, Nafsika Theodoropoulou, J. David Carey

**Affiliations:** 1 Joule Physics Laboratory, School of Computing Science and Engineering, University of Salford, Salford, M5 4WT UK; 2 Advanced Technology Institute, Faculty of Engineering and Physical Sciences, University of Surrey, Guildford, GU2 7XH UK; 3 Department of Electrical and Electronic Engineering, University of Surrey, Guildford, GU2 7XH UK

**Keywords:** Condensed-matter physics, Electronics, photonics and device physics

## Abstract

Er implanted Si is a candidate for quantum and photonic applications; however, several different Er centres are generated, and their symmetry, energy level structure, magnetic and optical properties, and mutual interactions have been poorly understood, which has been a major barrier to the development of these applications. Optically modulated magnetic resonance (OMMR) gives a spectrum of the modulation of an electron paramagnetic resonance (EPR) signal by a tuneable optical field. Our OMMR spectrum of Er implanted Si agrees with three independent measurements, showing that we have made the first measurement of the crystal field splitting of the ^4^I_13/2_ manifold of Er implanted Si, and allows us to revise the crystal field splitting of the ^4^I_15/2_ manifold. This splitting originates from a photoluminescence (PL) active O coordinated Er centre with orthorhombic C_2v_ symmetry, which neighbours an EPR active O coordinated Er centre with monoclinic C_1h_ symmetry. This pair of centres could form the basis of a controlled NOT (CNOT) gate.

## Introduction

Si is ubiquitous in information processing technology, whereas Er is ubiquitous in communications technology. The efficient generation and amplification of 1.5 μm radiation from Si would solve interface bottlenecks in telecommunication networks. To this end, the investigation of Er implanted Si has been pursued^1^, and although recent progress has been made in improving the efficiency^2^, lasing has not yet been demonstrated. Another potential application of Er implanted Si is for quantum technologies (QTs). The difficulty in obtaining lasing in Er implanted Si is of little consequence for QT applications since potential devices would involve the conversion of telecoms photons to spin states or carriers, and quantum devices would ultimately be based on individual Er atoms.

The processing technology of Si is decades ahead of any other material, and any quantum computing architecture developed in Si will move from lab demonstration to production far quicker than for any other material. The latest lithography tools can pattern 7 nm features for mass production Si integrated circuits, which is on the scale required for many quantum device architectures. Donor impurities, such as P and Bi (ref. ^3^), in Si are attractive for QTs because they could utilise existing Si processing technology, and because of long spin coherence times; however, they cannot be addressed at telecoms wavelengths with high efficiency. Successful addressing of qubits at telecoms wavelengths will allow integration with the fibre telecommunication network for secure quantum communication and allow small quantum computers to be linked into larger ones. Rare earth ions in Si offer an extra barrier to decoherence from the atomic shielding of the f-orbital by the 5 s and 5p shells. Quantum architectures developed using ion implantation and lithography would be fully compatible with conventional IC tooling, and are therefore scalable. Combining the shielding of Er f-electrons with the low nuclear spin and processing pedigree of Si, Er implanted Si offers a unique platform on which to implement QTs at telecoms wavelengths. Characterisation of Er implanted Si is therefore critical in identifying technologically useful optical centres and informing processing strategies to optimise these centres.

Optical characterisation of Er implanted Si presents a number of difficulties. The low absorption and emission cross-section of Er, low overall number of implanted ions, and low depth of implants (typically no more than ~2 μm), makes direct optical measurements, particularly absorption, extremely challenging. PL can be readily obtained by indirect excitation, where above band gap radiation generates excitons which are trapped by, and transfer their energy to, Er^3+^ centres from which radiative relaxation gives rise to characteristic emission at 1.5 μm (ref. ^1^). The crystal field splitting of the ^4^I_15/2_ ground state of Er implanted Si can be determined from PL measurements; however, the splitting of the ^4^I_13/2_ excited state remains elusive, with only the first two or three levels determined from so called “hot lines”, i.e. PL transitions from thermally populated crystal field states in the excited state manifold^4^. The difficulty in distinguishing hot lines from each other, and other PL lines, along with the requirement of cryogenic temperature for PL from Er implanted Si makes identification of higher lying ^4^I_13/2_ crystal field states from hot lines unlikely.

When co-implanted with O, Er generates a variety of centres that are detectable by PL and EPR. In addition, molecular beam epitaxy (MBE) grown Er doped Si generates PL lines similar to those observed in Er implanted Si and Zeeman measurement of this PL have been reported^5,6^. EPR measurements have identified a set of monoclinic and trigonal O coordinated Er centres^7–10^. PL measurements identify a cubic Si coordinated Er centre and at least one additional O coordinated Er centre with lower than cubic symmetry^4,11^, although the symmetry and energy level structure of this centre has not been fully identified. Zeeman measurements of MBE grown Er doped Si have identified an orthorhombic centre with proposed O and Si coordination, but this centre was not observed in EPR measurements of Er implanted Si. The properties of these centres are summarised in Table 1; however, the relationship between these centres is not currently understood. Further understanding of these centres will assist researchers in developing processing strategies to favour one particular type of centre. This would benefit both photonic and QT applications of Er implanted Si.

**Table 1:** The various centres in Er doped Si identified by EPR, PL and Zeeman measurements.

Centre label	Experimental method	Symmetry	Coordination	Ref.
OEr-1, OEr1′, OEr-3	EPR	Monoclinic C_1h_	O	^8–10^
OEr-2, OEr-2′, OEr-4	EPR	Trigonal C_3v_	O	^7–10^
Er-1	Zeeman	Orthorhombic C_2v_	O, Si	^5,6^
Er-C	PL	Cubic	Si	^4,11^
Er-O1, Er-O2	PL	Low	O	^4^

The OMMR technique we use in this study involves the modulation of the EPR signal from Er implanted Si by a tuneable laser resonant with an Er centre’s electron dipole transition. The OMMR technique can be thought of as the inverse of traditional optically detected magnetic resonance (ODMR) techniques, which involve the modulation of optical luminescence or absorption signals, by a microwave field. Whereas the OMMR technique involves the modulation of a microwave absorption signals by an optical field. Absorption based ODMR could, in principle, be used to probe the excited state of Er implanted Si. However, since absorption measurements are not feasible, neither would ODMR based on absorption.

In this work, we use OMMR to measure, for the first time, the crystal field splitting of the first excited state of Er implanted Si. We determine the energy level structure of a PL active O coordinated Er centre and identify it as having orthorhombic C_2v_ symmetry. We also report previously unreported PL lines from Er implanted Si. We show that the monoclinic EPR centre and orthorhombic PL centre are distinct, but highly localised centres. This pair of centres could be exploited for CNOT gates in an Er implanted Si based quantum computer architecture.

## Results and Discussion

### OMMR measurements

**Figure 1:**
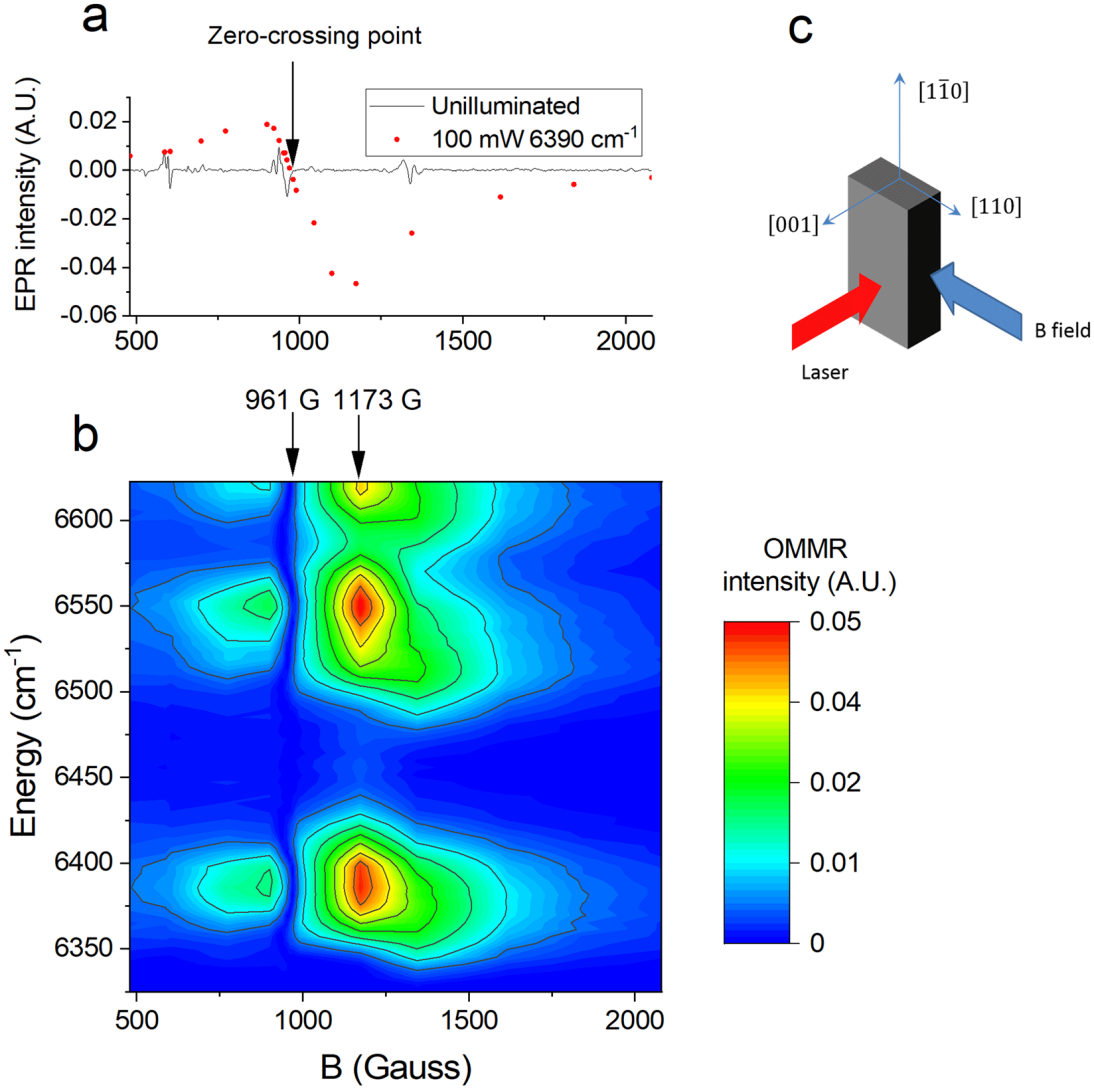
(**a**) EPR spectrum of 10^19^ cm^−3^ Er and 10^20^ cm^−3^ O implanted Si. The illuminated EPR spectrum was taken from the OMMR intensity at 6390 cm^−1^ from each of the OMMR spectra in Fig. 1(b); this corresponds to a coarse optically generated EPR spectrum. The OMMR intensity after the zero-crossing point was made negative to take into account that the second lock-in gives the absolute value of the modulated EPR signal. (**b**) Contour plot constructed from multiple OMMR spectra at magnetic fields between 480 and 2300 G. (**c**) Alignment of the sample for OMMR measurement showing the direction of laser irradiation and the magnetic field. The temperature was 10 K, the microwave frequency was 9.37 GHz for all measurements.

**Figure 2:**
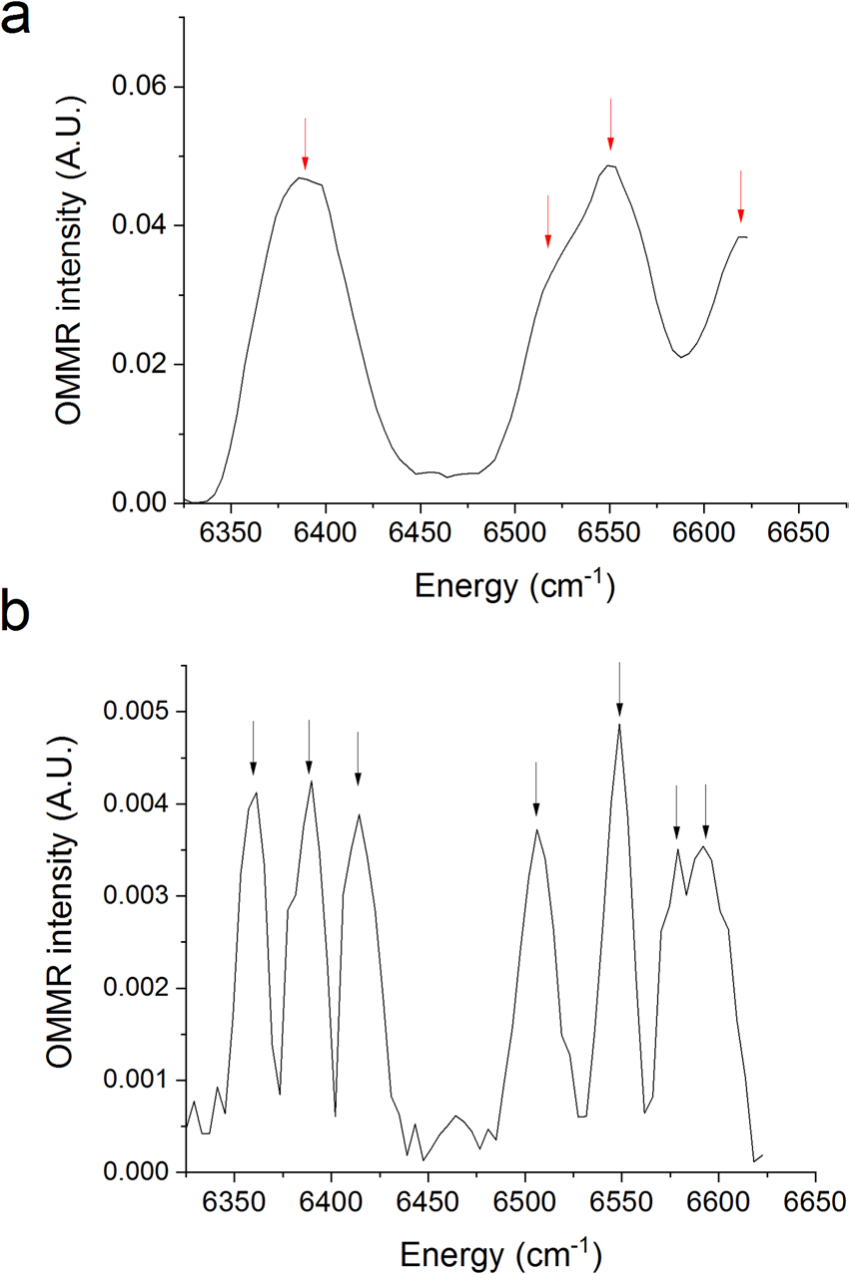
OMMR spectra of 10^19^ cm^−3^ Er and 10^20^ cm^−3^ O implanted Si taken at (**a**) 1173 G and (**b**) 961 G. Arrows indicate the identified peaks. The temperature was 10 K and the microwave frequency was 9.37 GHz.

Fig. 1(a) shows the EPR spectrum of Er implanted Si at 10 K, measured at a microwave frequency of 9.37 GHz. Three strong narrow resonances are visible at 595, 950 and 1330 G, with peak-to-peak widths of 6, 25 and 20 G, respectively. By comparisons to angular dependent EPR measurements made previously by Carey *et al*. on similar Er implanted Si samples^8–10^, we conclude that the resonance at 950 G originates from the OEr-1′ monoclinic centre, and the 595 and 1330 G resonances originate from either the OEr-1′ or OEr-3 monoclinic centres. Also shown is the EPR spectrum under 100 mW, 6390 cm^−1^ (1565 nm) laser irradiation which shows a broad strong EPR resonance that only occurs under laser irradiation, centred at ~970 G with a peak-to-peak width of ~320 G. Fig. 1(b) shows a contour plot constructed from many OMMR spectra taken at magnetic fields between 480 and 2300 G. The strong optically generated EPR resonance appears as two bands at 850 and 1170 G, since the second lock-in gives the absolute value of the modulated EPR signal. The sample alignment used in the OMMR measurement is shown in Fig. 1(c). The strongest OMMR spectrum, at 1173 G, is shown in Fig. 2(a). Four broad bands can be observed in laser energy centred at 6390, 6520, 6550 and 6620 cm^−1^, with full width at half maximums (FWHMs) of ~60 cm^−1^.

When the sample is rotated in the $(1\bar{1}0)$ plane, both the narrow EPR and broad optically generated resonances shift in magnetic field. By making small angle adjustments we were able to approximately align the zero-crossing point, at 961 G, of the broad optically generated resonance with the monoclinic EPR line at 950 G. At 961 G the OMMR spectrum switches from the broad spectrum to one with seven narrow peaks at 6360, 6389, 6415, 6506, 6548, 6579 and 6592 cm^−1^, with FWHM of ~15 cm^−1^, and reduces in intensity by an order of magnitude, as shown in Fig. 2(b). This weak narrow spectrum cannot be resolved in Fig. 1(b). At different sample orientations, when the zero-crossing point of the broad optically generated resonance does not correspond with a narrow EPR resonance, the OMMR spectrum is largely featureless, see Supplementary Fig. S1, demonstrating that the 961 G OMMR spectrum is related to the monoclinic EPR line at 950 G.

### Photoluminescence measurements

**Figure 3:**
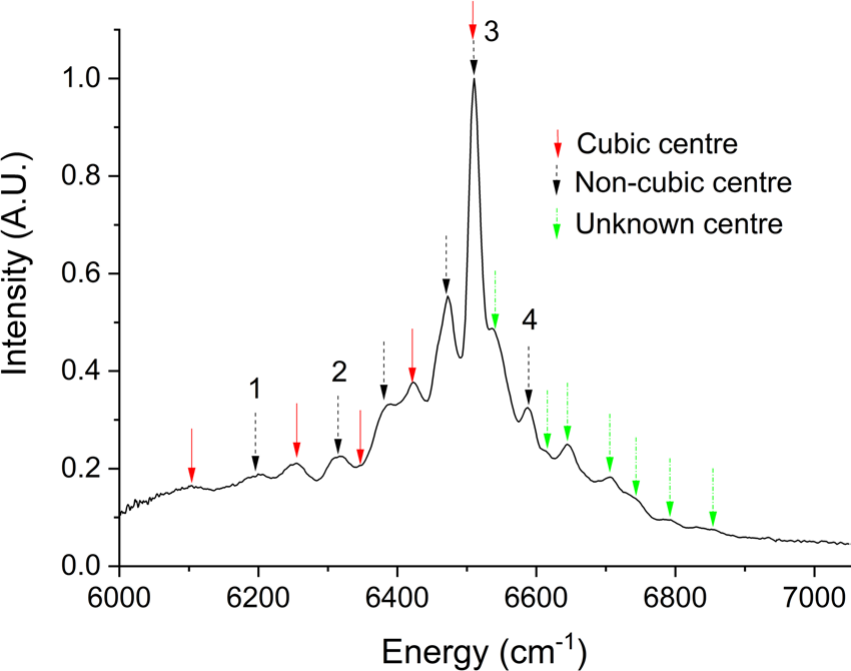
PL spectrum of 10^19^ cm^−3^ Er and 10^20^ cm^−3^ O implanted Si at 60 K. The arrows indicate the peaks corresponding to the various identified centres.

PL measurements at 60 K of the Er implanted Si sample are shown in Fig. 3. We identify ~17 peaks in this spectrum, all with FWHM ~20 cm^−1^. The PL peaks associated with a Si coordinated cubic Er centre (Er-C) have previously been unambiguously determined from PL measurements of Er implanted Si with low O concentrations^11,12^. We observe the same peaks in Fig. 3, identified with red arrows. With the inclusion of more oxygen atoms, either through O co-implantation^12^ or implantation of Er into CZ Si with high O impurities^4^, more PL peaks are observed. These peaks were attributed to an O coordinated Er centre with lower than cubic symmetry and an energy level structure of this low symmetry centre, named Er-O1, was proposed^4^. In the descriptions of both the Er-C and Er-O1 centres, peak 3 in Fig. 3 was assumed to be the crystal field ground state. Peaks with energy higher than peak 3 are not commonly observed in Er implanted Si. However, there is some evidence of peak 4 in Er implanted Si with similar processing conditions to ours^13^, but its significance was not discussed. The peaks marked with green arrows have not been reported previously, to the best of our knowledge, and the centre is unknown. We propose this is due to our sample being optimised for EPR rather than PL, and the high sensitivity of our PL system. Given that the peaks of the unknown centre have not been observed in Si with low O content, it is probably an O coordinated Er centre. The seven lines, if they are all from the same centre, indicate that it has lower than cubic symmetry. We attempted to fit the lines of the unknown centre with various sets of crystal field parameters (CFPs) for the common symmetries, but no unique fit was found. The energies of the PL peaks identified in Fig. 3 along with their assigned centre and symmetry are given in Supplementary Table S1, along with a comparison with the PL peaks identified in ref. ^4^.

We propose that the peaks observed in the OMMR spectra in Fig. 2(a,b) are an indirect measurement of the crystal field splitting of the ^4^I_13/2_ manifold, and that there are two distinctive types of OMMR spectra: a strong broad spectrum, as in Fig. 2(a), and a weak narrow spectrum, as in Fig. 2(b), which represent two different Er centres. To test this hypothesis, we performed crystal field analysis on the known energy splitting of the ^4^I_15/2_ manifold determined by PL to see if they predict the splitting observed in our OMMR spectra.

### Crystal field analysis

**Figure 4:**
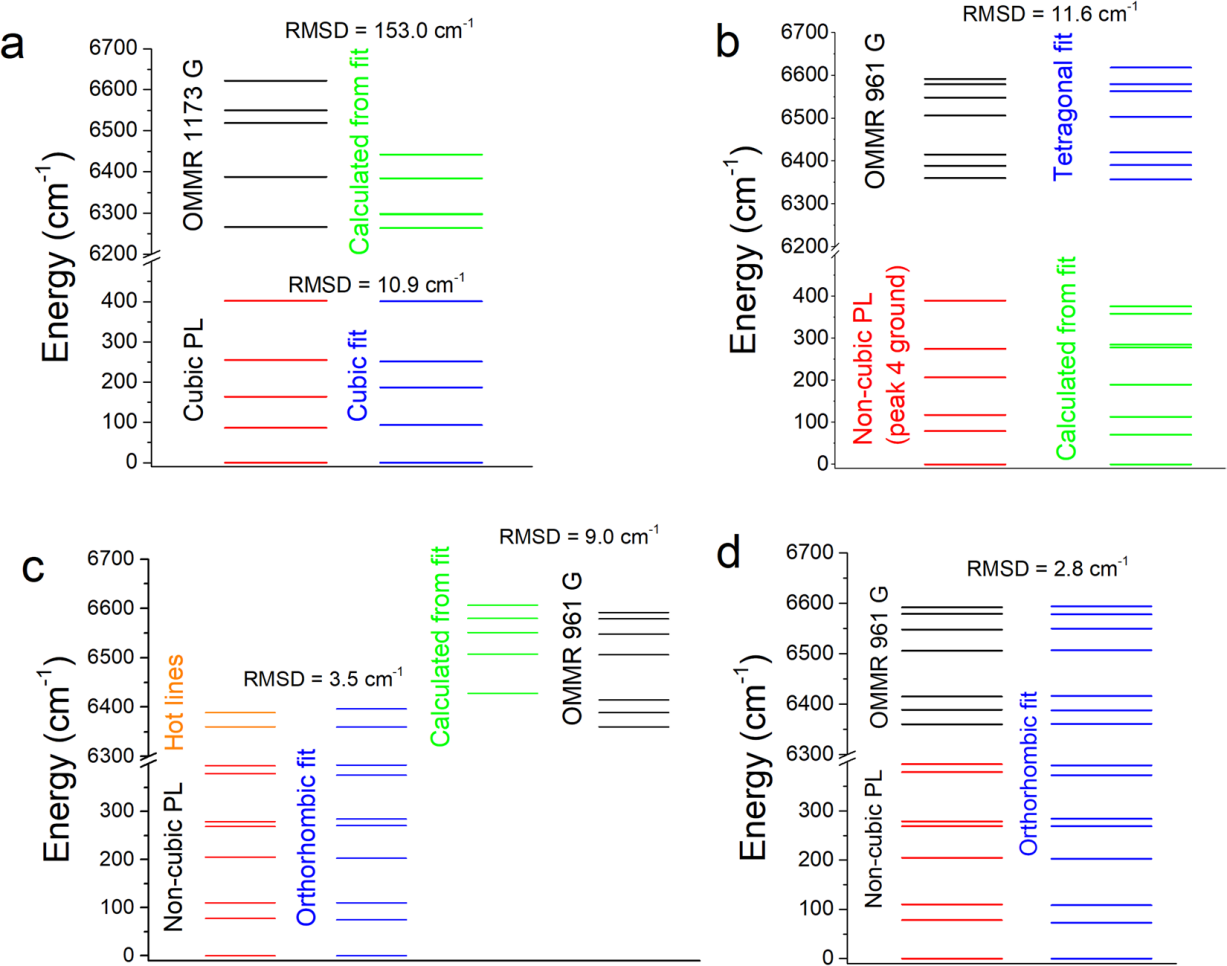
(**a**) The cubic energy levels from Fig. 3, and a fit to them assuming cubic (T_d_) symmetry, along with the calculated splitting of the ^4^I_13/2_ manifold, and the 1173 G OMMR lines. (**b**) Non-cubic energy levels from Fig. 3, assuming peak 4 is the crystal field ground state, with a tetragonal D_2d_ fit to the 961 G OMMR spectrum, and the calculated splitting of the ^4^I_15/2_ manifold. (**c**) An orthorhombic fit to our non-cubic PL energy levels and hot lines from the non-cubic centred identified by Przybylinska (ref. ^4^), and the calculated splitting of the rest of the ^4^I_13/2_ manifold along with the 961 G OMMR spectrum. (**d**) Orthorhombic fit to our non-cubic PL lines and 961 G OMMR spectrum. All measured and calculated splitting of the ^4^I_13/2_ manifold have been aligned with the 961 G OMMR spectrum to take into account the offset energy, E_off_ (see Methods).

Since all the reported narrow EPR resonances belong to low symmetry centres, we assume that the broad optically generated EPR resonance shown in Fig. 1(a), which has an OMMR spectrum shown in Fig. 2(a), originates from the Er-C cubic centre. To confirm this, we fitted a set of cubic crystal field parameter (CFPs) to the cubic PL lines identified in Fig. 3, which, as shown in Fig. 4(a), resulted in a very good fit: root mean square deviation (RMSD) = 10.9 cm^−1^. The fitted CFPs, were similar to those previously reported for the Er-C centre^4^. Using these fitted CFPs we calculated the splitting of the ^4^I_13/2_ manifold and compared it to the OMMR at 1173 G; however, there is no match (RMSD = 153.0 cm^−1^). We can therefore discount OMMR at 1173 G as originating from the Er-C centre.

The 961 G OMMR spectrum could originate from the Er-O1 centre, which has undetermined, lower than cubic symmetry^4^. It has the predicted seven lines for the splitting of the ^4^I_13/2_ manifold with lower than cubic symmetry. This could be the monoclinic symmetry of the OEr-1′ centre^8^, which has a resonance at around the same magnetic field, or the orthorhombic symmetry of the Er-1 centre identified by Zeeman measurements^5,6^. Applying the same procedure used for Er-C presented a problem. There are seven energy levels reported in the Er-O1 centre and five corresponding peaks that could initially be identified in our PL spectrum. This is not enough peaks to fit either the nine CFPs for orthorhombic symmetry, or fourteen CFPs for monoclinic symmetry. Analysis of *g* tensors indicate the monoclinic OEr-1′ centre can be approximated with a tetragonal field^14^. We attempted fitting a set of tetragonal D_2d_ CFP_S_ to the seven Er-O1 lines; however, the fit was poor, and the calculated splitting of the ^4^I_13/2_ manifold did not match the 961 G OMMR spectrum. We then tried fitting a set of tetragonal CFPs to the 961 G OMMR, and, as shown in Fig. 4(b), there was a good fit (RMSD = 11.6 cm^−1^). Using these tetragonal CFPs, we calculated the splitting of the ^4^I_15/2_ manifold, but these didn’t match our low symmetry PL peaks or the Er-O1 energy levels. However, if we assume that peak 4 in Fig. 3 is the ground state, there is a good match to our non-cubic PL if we also assume peaks 1 and 2 contain two closely spaced peaks, as shown in Fig. 4(b). We also note that the splitting of the first two levels of the ^4^I_13/2_ manifold in Er-O1, measured by PL hot lines, matches the splitting of the first two levels of the 961 G OMMR.

Given the information from the tetragonal fit in Fig. 4(b), we propose that peak 4 is the crystal field ground state and that if peaks 1 and 2 contain two levels we can revise our non-cubic PL energy level structure to that shown in Fig. 4(c). Along with PL hot lines from ref. ^4^, this gives a total of ten energy levels, which allows us to fit orthorhombic CFPs. There is an excellent fit (RMSD = 3.5 cm^−1^), and using these CFPs we can calculate the rest of the ^4^I_13/2_ splitting, which gives a very good match to the 961 G OMMR (RMSD = 9.0 cm^−1^). This accurate prediction of the OMMR spectrum from independent measurements shows conclusively that the 961 G OMMR spectrum is a measurement of the splitting of the ^4^I_13/2_ manifold of the non-cubic PL centre. Using all the non-cubic PL and 961 G OMMR lines allows us to refine the orthorhombic fit, as shown in Fig. 4(d). The CFPs for the orthorhombic fit in Fig. 4(d) are ${B}_{0}^{2}$ = 68, ${B}_{0}^{4}$ = 2081, ${B}_{0}^{6}$ = −1, ${B}_{2}^{2}$ = 152, ${B}_{2}^{4}$ = −200, ${B}_{4}^{4}$ = 252, ${B}_{2}^{6}$ = −270, ${B}_{4}^{6}$=−109, ${B}_{6}^{6}$ = −30 (cm^−1^). Fitting a set of monoclinic CFPs, which requires the same nine CFPs for an orthorhombic fit, plus and extra five^15^, to the experimental energy levels in Fig. 4(d) gave a similar RMSD as the orthorhombic fit, and the CFPs also contained in the orthorhombic fit were very similar. Therefore, we cannot use CFP fitting to distinguish between monoclinic and orthorhombic symmetry. The centre we have identified is similar to the Er-O1 centre identified by Przybylinska^4^, but we have revised the energy level structure. We refer to this revised Er-O1 centre as Er-O1R.

### OMMR mechanism

The mechanism for optical excitation of Er implanted Si is well established and involves the generation of carrier pairs by above band-gap irradiation. These excitons are then trapped by an Er-related defect^16^. Various trap states are known to form at energies between 10 and 510 meV below the conduction band. Following trapping, excitons can transfer their energy to Er ions located at the trap centres to excite them to the ^4^I_13/2_ state. These trap states are believed to impart n-type conductivity to Er implanted Si. We have confirmed that our sample is n-type by thermopower measurements and has a Seebeck coefficient of −1.096 ± 0.007 mV/K, see Supplementary Fig. S2. After implantation and annealing, the resistivity of the implanted layer decreased by around six orders of magnitude. A model developed for the equivalent thermopower of planar structures shows that the contribution of the bulk p-doped Si to the measured thermopower is negligible^17^.

**Figure 5:**
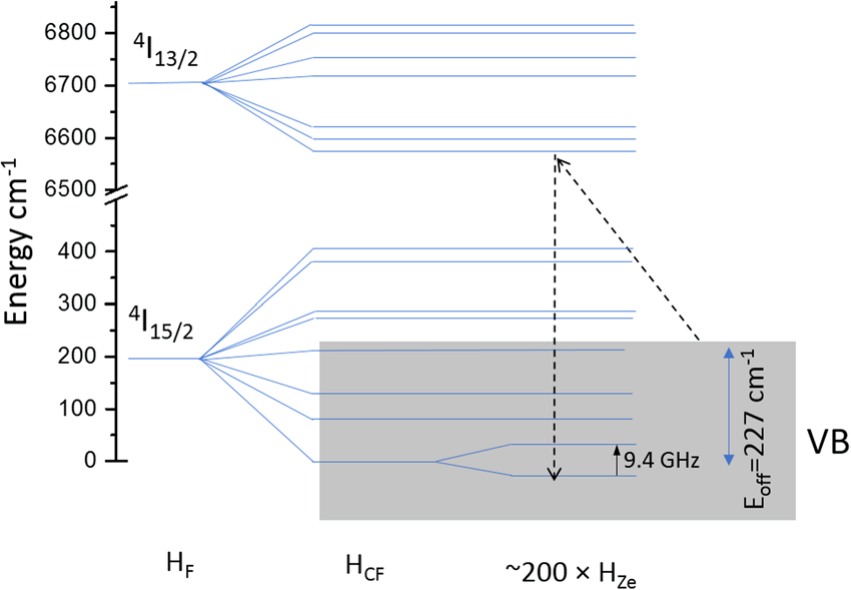
Energy level diagram showing a possible mechanism by which the OMMR signal is generated. H_F_, H_CF_ and H_Ze_ represent the free ion, crystal field and Zeeman splittings, respectively. The actual crystal field splitting is shown, the Zeeman splitting is only shown for the crystal field ground state for clarity.

To understand the mechanism that gives rise to the OMMR signal, we examined possible ways that absorption into the ^4^I_13/2_ manifold could increase the EPR signal. We can assume that at 10 K the EPR signal in our OMMR measurement arises from X-band microwave absorption in the Zeeman levels of the crystal field ground state of the ^4^I_15/2_ manifold, as shown in Fig. 5. Given a long enough spin-lattice relaxation time, T_1_, which is reasonable at 10 K, this Zeeman transition will readily saturate, so a modulation of the EPR signal can arise from repopulation of the Zeeman ground state. Since the OMMR spectrum shows the splitting of the ^4^I_13/2_ manifold, absorption into each ^4^I_13/2_ crystal field level will relax back to the ^4^I_15/2_ Zeeman ground state and increase the EPR signal. We know from the offset energy, see Methods, that this absorption must come from a level 227 cm^−1^ above the crystal field ground state. In rare earth doped semiconductors, the 4 f ground state manifold was previously assumed to lie deep within the valence band^18–20^, based partly on the separation of the 4f-levels of isolated rare earth ions from the vacuum level. We have recently made the first observation of direct optical transitions from the silicon conduction band to internal 4f-levels of implanted Ce, Eu, and Yb, which gave a significant enhancement of emission^21^. We also showed that their 4 f ground state manifolds lie ~1000 cm^−1^ above the valence band. This precedent of band state to 4f-level transitions indicates that transitions from the valence band to the ^4^I_13/2_ excited state of Er implanted Si are feasible, and may be a significant enhancement on intra 4 f transitions. We therefore propose a possible mechanism for the OMMR process, illustrated in Fig. 5, which involves optically induced transitions from the valence band to the ^4^I_13/2_ manifold, which subsequently relax to the ^4^I_15/2_ Zeeman ground state and enhance the EPR signal, with an optical spectral dependence matching the crystal field splitting of the ^4^I_13/2_ manifold. The offset energy E_off_, indicates that the ^4^I_15/2_ manifold is partially buried in the valence band.

At 10 K, relaxation from ^4^I_13/2_ to ^4^I_15/2_ should be almost entirely radiative, with a radiative relaxation time, τ_r_ ~ms. However, coupling to defect sates could cause non-radiative decay. We have measured the spin-lattice relaxation time of the monoclinic EPR centre, T_1Mo_, in a sample with a 3 × 10^17^ cm^−3^ Er concentration to be ~ms, see Supplementary Fig. S3, and we were able to measure EPR resonances from this sample. Since EPR from the orthorhombic Er-O1R centre can’t be measured, even from a sample with 1 × 10^19^ cm^−3^ Er, its spin-lattice relaxation time, T_1Or_, should be>>ms, in order for the Zeeman ground state to become saturated. Therefore, we expect τ_r_ <<T_1Or_, which would allow pumping of the Zeeman ground state by transitions from the ^4^I_13/2_ to manifold.

### *g* tensor calculation

The set of CFPs we have obtained from tetragonal, orthorhombic and monoclinic fitting can be used to calculate the expected EPR *g* tensors, see Methods. We would expect the calculated *g* tensors to match those of the OEr-1′ centre identified by Carey *et al*.^8^, since the OMMR measurement can only be observed at the magnetic field of a resonance corresponding to this centre. However, Table 2 shows the *g* tensors of all the Er centres identified from EPR measurements of Er implanted Si, those obtained from Zeeman measurements of MBE grown Er doped Si and our calculated *g* tensors. The EPR *g* tensors correspond only to the crystal field ground state of the ^4^I_15/2_ manifold, but Zeeman measurements were made of both the ^4^I_15/2_ and ^4^I_13/2_ manifold. There is a good match for the *g* tensors calculated from our tetragonal D_2d_ fit to the 961 G OMMR lines and to the Er-1 centre identified by Zeeman measurements. There is a possibility that residual defects could give rise to the OMMR signal; however, this agreement with another set of independent measurements confirms beyond any reasonable doubt the 961 G OMMR spectrum is a measurement of the Er ^4^I_13/2_ crystal field splitting and does not result from residual defects. The *g* tensors calculated from orthorhombic and monoclinic fits to our PL and OMMR lines are even closer to the Er-1 centre identified by Zeeman measurements of MBE grown Er doped Si, and do not match any of the EPR centres of Er implanted Si. This also shows that the Er-O1R and Er-1 centres are the same centre and this centre is a separate centre to any of the identified EPR centres. The symmetry of the Er-O1R centre should therefore also be same orthorhombic C_2v_ symmetry as the Er-1 centre. However, there must be a link between the Er-O1R centre and the OEr-1′ centre since the OMMR spectrum of the Er-O1R centre is only observed when at the EPR resonance of the OEr-1′ centre, this implies that microwave absorption, and hence population of the spin-up state of the OEr-1′ centre allows the OMMR mechanism of the Er-O1R centre to proceed. This indicates that the OEr-1′ and Er-O1R centres exist in close proximity, possibly as a dimer. This has important implications for QT applications of Er implanted Si, since single molecular magnets (SMMs) containing two weakly coupled rare earths with different coordination environments have been proposed as the basis of a two qubit CNOT gate^22^, and SMMs containing two Tb^3+^ ions have been shown to meet all the conditions needed for a universal CNOT gate^23^. The OEr-1′ and Er-O1R centres appear to be the solid-state analogue of these SMM systems but have all the device fabrication advantages of being based in Si. In addition, the strong axial anisotropy displayed in the *g* tensors of the OEr-1′ centre and, in particular, the Er-O1R centre are a requirement for the realisation of CNOT gates^22^. There are also implications for the photonic applications of Er implanted Si, where the ability to switch a 1.5 µm optical transition on with microwave or magnetic pulses could have applications in signal processing. We propose that the reason the Er-O1R centre is not EPR active is because the ^4^I_15/2_ crystal field ground state has a long T_1_, which is consistent with our model for the OMMR mechanism. This long T_1_ could make the EPR signal significantly weaker than centres with a shorter T_1_^24^. The dramatic difference in the *g* tensor of the OEr-1′ and Er-O1R centres is in line with previous findings that subtle differences in the structure of Dy SMMs can have dramatic effects on their magnetic properties^25^.

**Table 2:** Experimental and calculated g tensors of the crystal of field ground state of Er doped Si.

Centre	Manifold	Symmetry	g_x_	g_y_	g_z_	Ref.
**Experimental EPR**						
OEr-1	15/2	C_1h_	0.8	5.45	12.6	^8^
OEr-1′	15/2	C_1h_	0.8	5.45	12.55	^8^
OEr-3	15/2	C_1h_	1.09	5.05	12.78	^8^
OEr-4	15/2	C_3v_	2	6.23	6.23	^8^
OEr-2	15/2	C_3v_	0.45	3.46	3.22	^8^
OEr-2′	15/2	C_3v_	0.69	3.24	3.24	^8^
**Experimental Zeeman**						
Er-1	15/2	C_2v_	0	0	18.4	^6^
Er-1	13/2	C_2v_	0	0	14.8	^5^
**Calculated from fitted CFPs**						
Er-O1R	15/2	D_2d_	0	0	17.9	This work
Er-O1R	13/2	D_2d_	0	0	13.0	This work
Er-O1R	15/2	C_2v_	0	0	17.8	This work
Er-O1R	13/2	C_2v_	0	0	14.3	This work
Er-O1R	15/2	C_1h_	0	0	17.7	This work
Er-O1R	13/2	C_1h_	0	0	14.3	This work

## Conclusions

The OMMR spectrum of Er implanted Si at 961 G is accurately predicted by crystal field analysis of PL measurements, accurately predicts *g* tensors from Zeeman measurements, and agrees with PL hotline measurements. This agreement with three sets of independent measurements shows beyond any reasonable doubt that the 961 G OMMR spectrum is a measurement of the splitting of the ^4^I_13/2_ manifold, which represents the first measurement of the crystal field splitting of the ^4^I_13/2_ manifold of Er implanted Si. The OMMR spectrum originates from the Er-O1R centre and is only observed at a magnetic field which is at an EPR resonance of the OEr-1′centre, which indicates that these two centres are in close proximity. The Er-O1R centre is not observed in EPR measurements, which we propose is due to a longer T_1_ than the OEr-1′ and other EPR active centres.

An energy offset in the OMMR spectrum, compared to what would be expected from a direct absorption measurement, indicates that the OMMR signal originates from transitions from the top of the valence band, and that the ^4^I_15/2_ manifold is partially buried in the valence band. Because the OMMR mechanism involves transitions from the valence band, it may be restricted to rare-earth doped semiconductors, it may also requires a spin T_1_ long enough to allow saturation of the Zeeman transition. However, the OMMR technique could be used by other researchers investigating rare-earth doped semiconductors.

## Methods

### Ion implantation and annealing

The sample was prepared by implanting Er and O into P-doped < 100 > 500 µm thick Si wafer supplied by Topsil at 77 K. The unimplanted wafer had a measured resistivity of 8000 ± 500 Ωcm, corresponding to a P concentration of 5.5 ± 0.3 × 10^11^ cm^−3^. A range of implant energies was used to give a flat ion concentration profile down to a depth of around 1.5 µm, as illustrated in Supplemetary Fig. S4. Doses were chosen to give Er and O concentrations of 10^19^ and 10^20^ cm^−3^, respectively. Isotope specific implantation was used so that only the zero nuclear spin ^166^Er was implanted. After implantation the sample was annealed at 450 °C for 30 min to smooth the crystalline-amorphous interface, then at 620 °C for 180 min to recrystallize the amorphized region then at 850 °C for 30 s to activate the Er for EPR measurments. It was found that annealing at 850 °C significantly increased the EPR signal strength. This is in contrast to previous work where the same smoothing and recrystallization anneal was used, but the activation anneal was 900 °C for 30 s.^8–10^ This is probably due to inconstancies between different annealing furnaces. The relative EPR signal intensities for different activation annealing conditions are shown in Supplemetary Fig. S5.

### EPR and OMMR measurements

EPR measurements were taken on a Brucker EMX EPR spectrometer, incorporating a super high-Q resonator with optical access. The field modulation was 100 kHz, and the microwave frequency was 9.37 GHz. EPR measurements were recorded at various magnetic field directions approximately parallel to the [110] direction of the Er implanted Si sample with a tolerance of ±5°. For OMMR measurements we used the same spectrometer as for EPR measurements. Here, the output from an external cavity laser tuneable from ~1490 to 1620 nm, with an output power of up to ~20 mW, was fed into a C-band erbium doped fibre amplifier with a gain bandwidth of ~1510–1600 nm and an output of up to ~150 mW. The output of the fibre was collimated and then passed through a linear polarizer, parallel to $[1\bar{1}0]$ direction of the Er implanted Si sample, before being modulated with a mechanical chopper at ~30 Hz. The EPR signal output from the EMX EPR spectrometer on-board lock-in amplifier was fed into the input of a SRS830 lock-in amplifier referenced to the mechanical chopper. OMMR spectra were generated by sweeping the external cavity laser wavelength and reading the SRS830 lock-in signal to give a spectrum of EPR signal that has been modulated by the laser, ΔEPR(λ). A microwave power of 2.1 mW and a time constant of 5 ms was used for OMMR measurements.

### PL and electrical measurements

PL spectra were obtained by placing the sample in a cold finger LN_2_ cryostat at 60 K, dispersing the fluorescence generated by a 462 nm 50 mW laser diode in a Bentham TMc300 monochromator, with a resolution of 3 nm, and detecting with an IR PMT coupled with standard phase sensitive detection. All spectra were corrected for the system response. Thermopower and conductivity measurements were carried out using a method described previously^26^.

### Crystal field analysis

The Hamiltonian (H) of the Er^3+^ in our OMMR measurement can be described as

1 $$H={H}_{F}+{H}_{CF}+{H}_{Ze}$$

*H*_*F*_ accounts for the interactions that occur in a free Er ion. There are many interactions thought to occur in the free ion, these can be broken down as follows^27^.

2 $${H}_{F}={H}_{0}+{H}_{ee}+{H}_{SO}+\mathop{\sum }\limits_{i=1}^{4}\,{H}_{i}(corr)$$

Where *H*_0_is a constant representing the kinetic energy of the f electrons and their coulomb interactions with the nucleus and electrons in filled shells. *H*_*ee*_ and *H*_*SO*_ represent electron-electron intra-shell coulomb and spin-orbit interactions, respectively. $\mathop{\sum }\limits_{i=1}^{4}{H}_{i}(corr)$ are a set of four corrective terms which include two and three body operators. Together, these give 20 parameters to represent *H*_*F*_. Each rare earth has its own set of *H*_*F*_ parameters, and these vary little between hosts. We used those given by Carnall *et al*. for Er:LaF_3_^28^.

*H*_*CF*_ describes a perturbation generated by ligands of the host crystal lattice surrounding the Er^3+^ ion. The multipole expansion of *H*_*CF*_ is defined as the linear combination of a set of spherical tensors, ${C}_{q}^{(k)}$, and structural factors, ${B}_{q}^{k}$, which are referred to as crystal field parameters and represent the symmetry of the environment^29^.

3 $${H}_{CF}=\sum _{k,q}\,{B}_{q}^{k}{C}_{q}^{(k)}$$

Details of the construction of *H*_*CF*_ are given elsewhere^30^. Each site symmetry of Er^3+^ has its own particular set of non-vanishing CFPs.

The Zeeman interaction, *H*_*Ze*_, is given by

4 $${H}_{Ze}={g}_{J}{\mu }_{B}{\boldsymbol{J}}.{\boldsymbol{H}}$$

Where *g*_*J*_ is the Landé factor, *μ*_*B*_ is the Bohr magneton, ***J*** is the angular momentum operator, and ***H*** is the magnetic field strength^31^. ***H***_***Z****e*_ was around two orders of magnitude smaller than the crystal field widths. This meant Zeeman splitting was not observed on the OMMR spectra, and was therefore not considered during the fitting procedure. To fit OMMR and PL lines, differences between the eigenvalues of *H* and experimental energy levels were minimised with a least squares fitting algorithm.

### Offset energy

At cryogenic temperatures, and if direct intra-manifold transitions are being considered, the highest energy PL transition between two manifolds in a rare earth ion represents the energy separation of the crystal field ground states of the manifolds. Similarly, the lowest energy absorption transition between the same two manifolds represents the same energy separation. The absolute energies of the OMMR and PL measurements are therefore highly significant for our model. From Fig. 3, the highest energy PL peak of the Er-O1R center is at 6587 cm^−1^, whereas the lowest energy transitions for the 961 G OMMR spectrum in Fig. 2(b) is at 6360 cm^−1^. If we assume the 961 G OMMR spectrum represents the splitting of the first excited state manifold, it must originate from a state that is an offset energy, E_off_, above the crystal field ground state of the ^4^I_13/2_ ground state manifold, where E_off_ = 6587–6360 = 227 cm^−1^. This is also important for our crystal field analysis since the inter-manifold separation calculated by *H*_*F*_ is only applicable to direct inter-manifold experimentally observed transitions. Therefore, for our crystal field analysis, E_off_ was added to the peak energies of the 961 G OMMR spectrum in Fig. 2(b).

### *g* tensor calculation

Each crystal field doublet has two sets of eigenvectors: $|\,+\,\rangle$ and $|\,-\,\rangle$. One for each pair of degenerate eigenvalues. The diagonal components of the *g* tensor, g_x_, g_y_, g_z_, are calculated using the first order perturbation expressions^31^.

5 $${g}_{x}=2{g}_{J}\langle \,+\,|{{\boldsymbol{J}}}_{x}|\,-\,\rangle ,\,{g}_{y}=2{g}_{J}\langle \,+\,|{{\boldsymbol{J}}}_{y}|\,-\,\rangle ,\,{g}_{z}=2{g}_{J}\langle \,+\,|{{\boldsymbol{J}}}_{z}|\,+\,\rangle $$

Where ***J***_***x***_, ***J***_***y***_, ***J***_***z***_, are the vector components of ***J***, such that ${{\boldsymbol{J}}}^{2}={{\boldsymbol{J}}}_{{\boldsymbol{x}}}^{2}+{{\boldsymbol{J}}}_{{\boldsymbol{y}}}^{2}+{{\boldsymbol{J}}}_{{\boldsymbol{z}}}^{2}$.

## Supplementary information

Supplementary information

## Data Availability

The datasets generated during the current study are available in the Mendely Data repository, 10.17632/5g67t8gsbc.1.
