# Supplementary material for: Optically modulated magnetic resonance of erbium implanted silicon
Source: Sci Rep. 2019 Dec 13;9:19031. doi: 10.1038/s41598-019-55246-z (PMC6910921; doi:10.1038/s41598-019-55246-z)
Supplement: Supplementary file 1 — Supplementary information [file 41598_2019_55246_MOESM1_ESM.pdf]

Supplementary Information

Optically modulated magnetic resonance of erbium implanted silicon

Mark A. Hughes[1], Heqing Li[1], Nafsika Theodoropoulou[1], J. David Carey[2,3]

[1]Joule Physics Laboratory, School of Computing Science and Engineering, University of Salford, M5 4WT, UK

[2]Advanced Technology Institute, Faculty of Engineering and Physical Sciences, University of Surrey, Guildford, GU2 7XH, UK

[3]Department of Electrical and Electronic Engineering, University of Surrey, Guildford, GU2 7XH, UK

**Correspondence should be addressed to: M.A.H (m.a.hughes@salford.ac.uk)*

Supplementary Table S1 PL peak energies, with their assigned centre and symmetry, compared and matched with those identified previously.

**Peak (cm^{-1})**	**Centre name**	**Symmetry**	**ref**	**Peak (cm^{-1})**	**Centre name**	**Symmetry**	**ref**
6100	Er-C	T_d	This work	6087	Er-C	T_d	[1]
6187	Er-O1R	C_{2v}	This work	6174	Er-O1	low	[1]
6199	Er-O1R	C_{2v}	This work				
				6230	Er-O1	low	[1]
6252	Er-C	T_d	This work	6256	Er-C	T_d	[1]
6311	Er-O1R	C_{2v}	This work				
6316	Er-O1R	C_{2v}	This work	6315	Er-O1	low	[1]
6346	Er-C	T_d	This work	6348	Er-C	T_d	[1]
6381	Er-O1R	C_{2v}	This work	6385	Er-O1	low	[1]
6422	Er-C	T_d	This work	6426	Er-C	T_d	[1]
				6438	Er-O1	low	[1]
6472	Er-O1R	C_{2v}	This work	6473	Er-O1	low	[1]
6510	Er-C	T_d	This work	6505	Er-C	T_d	[1]
6510	Er-O1R	C_{2v}	This work	6508	Er-O1	low	[1]
6536	Unknown	Unknown	This work				
6587	Er-O1R	C_{2v}	This work				
6614	Unknown	Unknown	This work				
6645	Unknown	Unknown	This work				
6706	Unknown	Unknown	This work				
6744	Unknown	Unknown	This work				
6792	Unknown	Unknown	This work				
6830	Unknown	Unknown	This work				

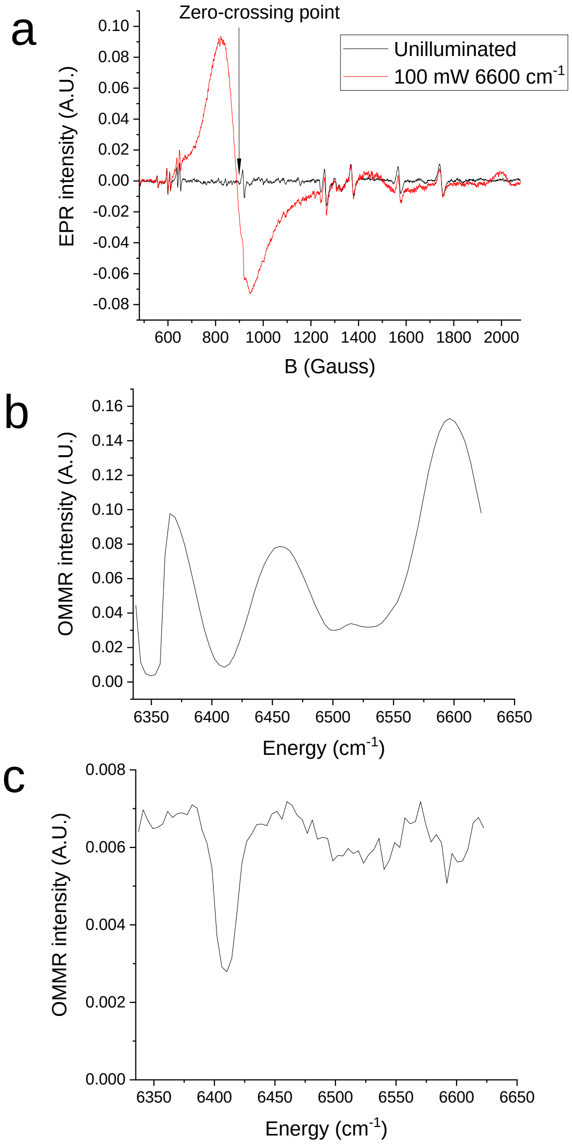

Supplementary Figure S1. (a) EPR spectrum of 10^{19} cm^{-3} Er and 10^{20} cm^{-3} O implanted Si sample rotated ~ 5° in the $(1\bar{1}0)$ plane so that the zero-crossing point avoids the OEr-1' EPR line. OMMR spectra of 10^{19} cm^{-3} Er and 10^{20} cm^{-3} O implanted Si taken at (b) the OMMR signal maximum at 826 G, and (c) the zero-crossing point at 887 G. The 887 G OMMR spectrum is flat, but contains a dip at 6410 cm^{-1}, which corresponds with a dip in the 826 G OMMR. The temperature was 8 K, the microwave frequency was 9.37 GHz.

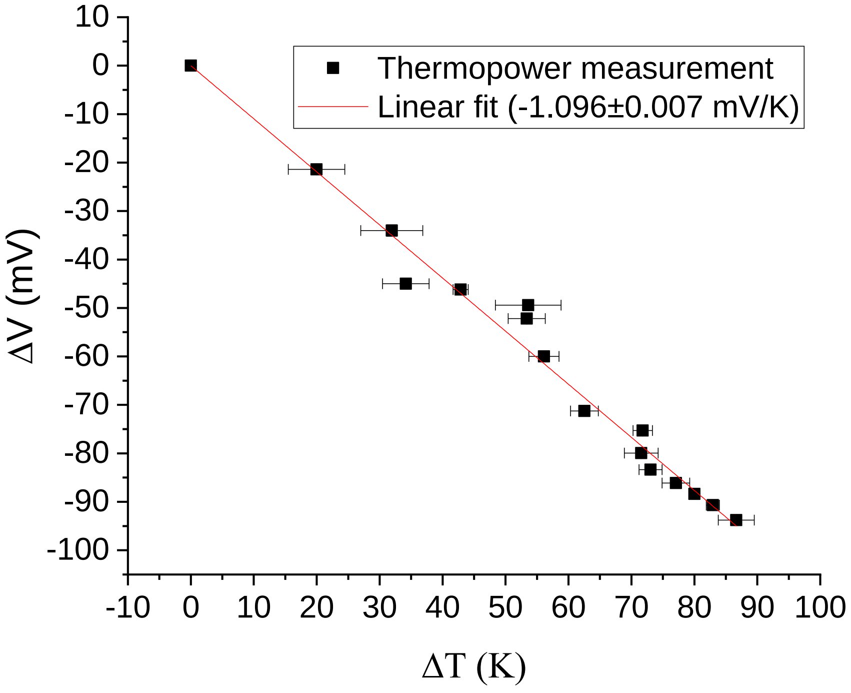

Supplementary Figure S2 Thermopower measurement of 10^{19} cm^{-3} Er and 10^{20} cm^{-3} O implanted Si. The Seebeck coefficient was independent of temperature and a linear fit to the thermopower measurements gave a Seebeck coefficient of -1.096±0.007 mV/K.

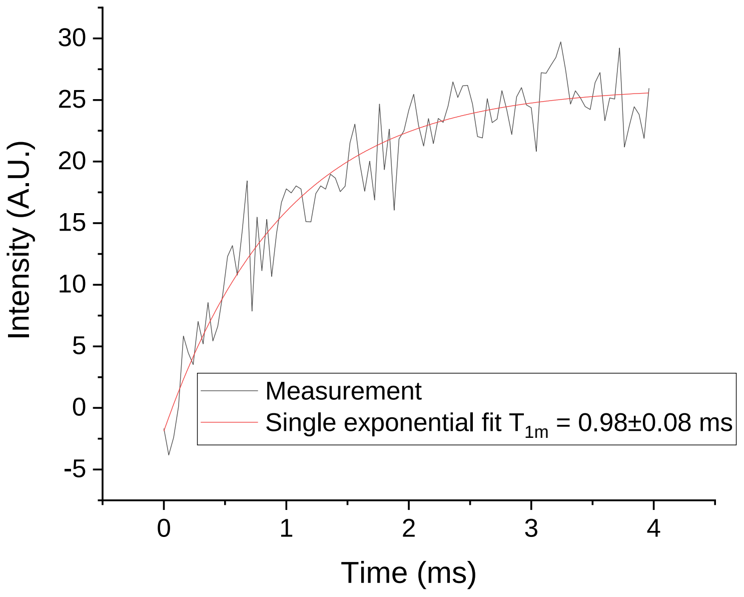

Supplementary Figure S3 Spin-lattice relaxation of the monoclinic OEr-1' EPR centre at 5 K in a sample with 3×10^{17} cm^{-3} Er and 10^{20} cm^{-3} O

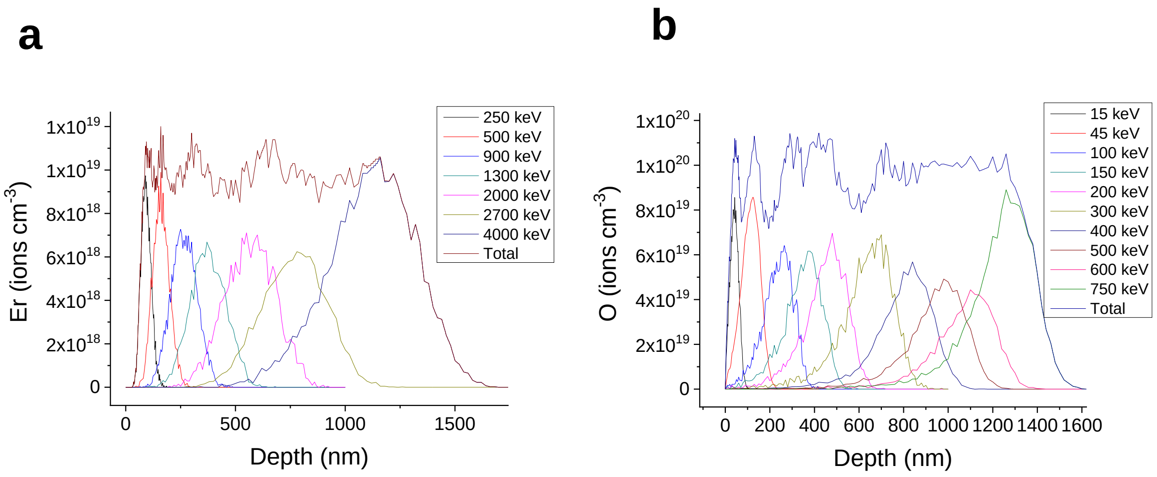

Supplementary Figure S4. (a) Simulated implant profile for Er with a peak concentration of 10^{19} cm^{-3} and a total areal dose of 2.6×10^{15} cm^{-2}. (b) Simulated implant profile for O with a peak concentration of 10^{20} cm^{-3} and a total areal dose of 1.3×10^{16} cm^{-2}.

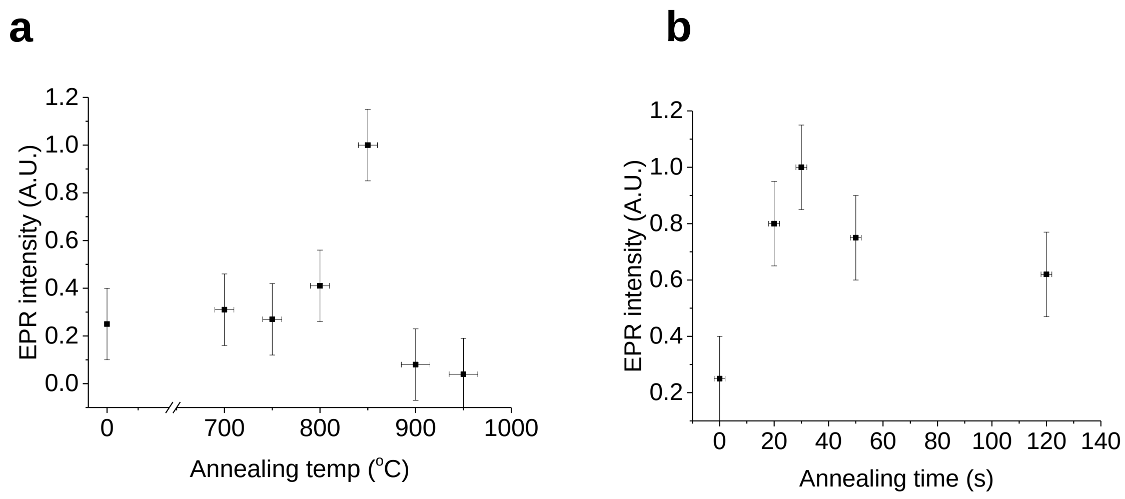

Supplementary Figure S5. EPR signal intensity for 10^{19} cm^{-3} Er and 10^{20} cm^{-3} O implanted Si after annealing at 450 °C for 30 min, then 620 °C for 3 hr, as a function of (a) activation annealing temperature with a time of 30s and (b) activation annealing time with a temperature of 850 °C.

References

1. Przybylinska H*, et al.* Optically active erbium centers in silicon. *Phys Rev B* **54**, 2532-2547 (1996).
